# Increasing Access to Family Planning Choices Through Public-Sector Social Franchising: The Experience of Marie Stopes International in Mali

**DOI:** 10.9745/GHSP-D-17-00011

**Published:** 2017-06-27

**Authors:** Judy Gold, Eva Burke, Boubacar Cissé, Anna Mackay, Gillian Eva, Brendan Hayes

**Affiliations:** a Independent Consultant, Melbourne, Australia.; b Independent Consultant, London, England.; c Marie Stopes International Mali, Bamako, Mali.; d Marie Stopes International, London, UK.; e Marie Stopes International, Washington, DC, USA.

## Abstract

**Background:**

Mali has one of the world's lowest contraceptive use rates and a high rate of unmet need for family planning. In order to increase access to and choice of quality family planning services, Marie Stopes International (MSI) Mali introduced social franchising in public-sector community health centers (referred to as CSCOMs in Mali) in 3 regions under the MSI brand BlueStar.

**Program Description:**

Potential franchisees are generally identified from CSCOMs who have worked with MSI outreach teams; once accredited as franchisees, CSCOMs receive training, supervision, family planning consumables and commodities, and support for awareness raising and demand creation. To ensure availability and affordability of services, franchisees are committed to providing a wide range of contraceptive methods at low fixed prices.

**Methods and Results:**

The performance of the BlueStar network from inception in March 2012 until December 2015 was examined using information from routine monitoring data, clinical quality audits, and client exit interviews. During this period, the network grew from 70 to 135 franchisees; an estimated 123,428 clients received voluntary family planning services, most commonly long-acting reversible methods of contraception. Franchisee efficiency and clinical quality of services increased over time, and client satisfaction with services remained high. One-quarter of clients in 2015 were under 20 years old, and three-quarters were adopters of family planning (that is, they had not been using a modern method during the 3 months prior to their visit).

**Conclusion:**

Applying a social franchising support package, originally developed for for-profit private-sector providers, to public-sector facilities in Mali has increased access, choice, and use of family planning in 3 regions of Mali. The experience of BlueStar Mali suggests that interventions that support quality supply of services, while simultaneously addressing demand-side barriers such as service pricing, can successfully create demand for a broad range of family planning services, even in settings with low contraceptive prevalence.

## Abstract (precis)

While social franchising has been highly successful with private-sector providers, in Mali the approach was expanded to public-sector community health clinics. From 2012 to 2015, these clinics served >120,000 family planning clients, 78% of whom chose long-acting reversible methods. Many clients were young, poor, and had not been using a method during the 3 months prior to their visit.

## INTRODUCTION

Mali has one of the world's lowest contraceptive prevalence rates (CPRs), with just 10% of married women reporting use of a modern method of family planning in the most recent Demographic and Health Survey (DHS) in 2012/13, below the West and Central Africa average of 17%.^1^^,^^2^ Use of contraceptives in Mali is much higher in urban than rural areas, with 23% of married women in the capital region of Bamako using a modern method compared with 3%–11% in other areas. A quarter of married women and 55% of sexually active unmarried women report an unmet need for family planning.^2^ The fertility rate in Mali remains high at 6.1 children per woman, along with high maternal mortality at 368 deaths per 100,000 live births.^1^

Along with low CPR, the contraceptive method mix in Mali remains heavily skewed toward short-acting contraceptives, with injectables and oral contraceptive pills the most commonly used methods.^1^ The majority of married women (72%) report obtaining their method from the public sector, most commonly community health centers, while 23% obtain their method from the private sector.^1^ Although family planning is included within the minimum package of health care services in the public sector, this does not always translate into availability of all methods. For example, many facilities do not have the commodities and equipment to provide long-acting or permanent methods. Even when these methods are available, the low rate of provision is a risk to provider competency and the safety of some family planning services.

Clinical social franchising—defined as the organization of small independent health care businesses into quality-assured networks^3^—can increase access to family planning services as well as choice and quality of services.^4^ Typically clinical social franchising involves intensive capacity building and training of private providers, which generally includes clinical training, branding, monitoring of quality, and commodity support, as well as support for marketing and consumer behavior change.^4^

Clinical social franchising can increase access to family planning services and to choice and quality of services.

Mali operates a “semi-public” approach at the lowest tier of its public health system (Box), thus presenting an opportunity to trial the introduction of the Marie Stopes International (MSI) BlueStar social franchising model into the public sector. This article describes the operation and results of the MSI Mali BlueStar network for its first 4 years of operation, using routine monitoring data, clinical quality audits, and client exit interviews. The MSI experience in Mali provides a useful case example of whether and how clinical social franchising approaches can be introduced in the public sector to improve family planning access, choice, and quality.

Mali provided an opportunity to trial the introduction of the social franchising model into the public sector.

BOX **Mali Health System**

Mali has a 4-tiered public health system, from community health centers to national referral hospitals. At the “entry-level” tier are the Centres de Santé Communautaires (CSCOMs), community health centers that are contracted by the government to provide a minimum package of basic health services. CSCOMs are the most common facilities for clients' first visits when seeking a health consultation.^5^

CSCOMs may be staffed by a single health care provider or a number of providers, including *matrones* (traditional birth attendants), nurses, pharmacy manager, midwives, and/or doctors. The majority of CSCOMs, particularly those in rural areas, are led by nurses rather than doctors.^5^^,^^6^ The government is responsible for training and supervision of all CSCOM staff although some training may be supported by external organizations with particular areas of expertise. Staffing levels and technical capacity vary considerably from one CSCOM to another.

Each CSCOM is established through an agreement with a commune mayor (the elected leader). The agreement specifies the shared responsibilities and financial commitments of each party and the devolving authority of the facility to the community. Management of the facility becomes the responsibility of an Associations de Santé Communautaire (ASACO), community health associations made up of individuals elected from the community.

The government provides most of the funding for initial CSCOM construction and equipment and for most health worker salaries. The remainder of CSCOM operating costs (e.g., some staff salaries, medication purchases, travel expenses, demand generation) are covered through patient fees for consultations and prescriptions, as well as contributions from the community.

Although CSCOMs themselves are private nonprofit entities, they exist to provide health services on behalf of the government and are thus commonly considered part of the public sector. In addition to public-sector health services, there are multiple private medical practices and hospitals in Mali, predominantly located in the urban areas of Bamako.

## PROGRAM DESCRIPTION: PUBLIC-SECTOR FRANCHISING MODEL IN MALI

Marie Stopes International Mali (MSIM) is the largest nongovernment provider of family planning services in Mali, providing family planning services in 7 of the country's 9 regions. In 2012, MSIM began supporting the provision of family planning services at 70 community health centers (known in Mali as *Centres de Santé Communitaires*, or CSCOMs), deploying a social franchise approach using the MSI global franchising brand BlueStar. CSCOMs were identified as the facility type with the most potential to build a franchise network focused on expanding access and choice, due to their wide geographical and population reach, compared with the limited presence and affordability of private facilities.

Initially the MSIM BlueStar network was launched in Koulikoro and Mopti, 2 regions with high levels of poverty and low CPR. In 2015, the network was expanded to the region of Sikasso, bordering Burkina Faso. Mopti is located in the north of Mali and has been affected since 2012 by the ongoing armed conflict between the government and Islamist insurgents.

The introduction of social franchising approaches to CSCOMs was part of the wider MSIM strategy to address differing capacity and needs across the Mali health system to deliver family planning services. In areas where family planning knowledge, use, and public capacity for provision of family planning service is low, MSIM provides mobile outreach services at CSCOM and other sites to increase awareness of, access to, and choice of family planning services. Once community awareness and demand for family planning has been primed, and CSCOMs with an interest and potential capacity to provide these services have been identified, MSIM shifts to a social franchising approach in the area, building CSCOM capacity to ensure sustained family planning choice and access. The role of mobile outreach teams in these areas is then reduced, such that these teams then focus nearly exclusively on the provision of permanent methods of family planning (described in more detail below). The complementary nature of services provided by franchisees and mobile outreach teams maximizes the range of family planning choices available while ensuring the quality of more clinically demanding services such as bilateral tubal ligation.

Once demand for family planning has been primed through mobile outreach services, MSIM shifts to a social franchising approach in the area.

Social franchising in Mali moves beyond capacity building of providers as it importantly includes the development of a long-term relationship with the *Association de Santé Communautaire* (ASACO) (Community Health Association) and the CSCOM. This involves quality improvement interventions and supply chain for contraceptives, as well as branding, local awareness creation, and contractual agreement on standards for service pricing (which includes price subsidies).

### Selection and Accreditation of Franchisees

Potential BlueStar franchisees may be identified from CSCOMs who are already working alongside MSIM mobile outreach teams. Other CSCOMs may be selected in consultation with the regional and district health authorities. Privately owned practices are also eligible to join as franchisees, but the limited scale and reach of the private sector in Mali has meant a limited focus on for-profit private providers.

Once identified, potential franchisees undergo an accreditation process to ensure their capacity to deliver quality family planning services. The accreditation process is conducted by MSIM and the district and regional health authorities and involves assessment of human resource capacity, current provision of family planning services, availability of materials and equipment, the building condition, and for CSCOMs, the functionality of the managing ASACO. A contract outlining the roles and responsibilities is signed between MSIM and the responsible ASACO (on behalf of the CSCOMs) or the clinic owner (for privately owned practices).

Once accredited, franchisees pay an annual membership fee of CFA 10,000 (US$17), which is always paid in practice. In return, franchisees receive:

- **Competency-based training, including refresher training,** in family planning counseling and services including provision of long-acting reversible contraception, infection prevention, HIV counseling and testing, emergency preparedness, stock management, and reporting
- Ongoing routine **clinical supervision and quality assurance,** including establishment of clinical minimum standards, quarterly supportive supervision visits (often joint missions with regional health authorities), and annual clinical audits
- **Consumables and commodities** to provide a wide range of quality family planning services
- **Marketing support** to raise community awareness of family planning services (see details in section below)
- **Branding** of health centers

Franchisees pay an annual membership fee, for which they receive in turn competency-based training, clinical supervision and quality assurance, consumables and commodities, marketing support, and branding of health centers.

### Franchisee Services

All social franchisees provide the following services under the BlueStar brand:

- Family planning counseling
- Provision of short-acting methods of family planning—pills, condoms, injectables, emergency contraception
- Provision and removal of long-acting reversible methods of family planning—the 10-year copper intrauterine device (IUD) and the 5-year implant
- HIV counseling and testing
- Referrals and appointments for voluntary female sterilization (MSIM does not currently offer vasectomy services)

Depending on the location of the client, clients choosing a permanent method are either referred to the MSIM clinics in Bamako or Mopti (if the CSCOMs are close enough, e.g., those in peri-urban areas in Bamako) or to the MSIM mobile outreach teams. CSCOMs have the monthly schedule of the mobile outreach teams and inform the client to return on the specific dates of the next outreach team visit to the CSCOM. The monthly schedule of the mobile outreach teams is also available to clients via posters in CSCOMs, the MSIM social marketing agents (described below), and the MSIM call center.

#### Pricing Strategy

As one of the intentions of the BlueStar network in Mali is to provide quality **family planning services at affordable costs**, MSIM purchases the family planning commodities through the public health system at a subsidized cost and provides them for free to BlueStar franchisees, who are only allowed to charge for these up to a maximum, subsidized price. This strategy enables franchisees to charge the same price as non-franchised CSCOMs for short-acting methods, and lower prices for long-acting reversible methods. For example, franchisees can provide IUDs for CFA 300 (US$0.49) compared with CFA 4,000–7,500 (US$6.60–12.30) at non-franchised CSCOMs. In line with the contract signed with the ASACOs, all BlueStar CSCOMs provide services free of charge to those unable to pay, and they may also run periodic promotional days with free services.

MSIM purchases family planning commodities through the public health system at a subsidized cost and provides them for free to franchisees.

### Awareness Raising

A range of strategies are used to increase community awareness and demand for a broad mix of family planning services. To directly support franchisees, MSIM employs social marketing agents to provide franchisee marketing and communication support; each agent supports 6 to 9 franchisees. Social marketing agents work closely with community health workers, providers, community and religious leaders, women's groups, and schools to conduct awareness raising and community mobilization activities. These involve group and one-to-one communication in a variety of locations such as schools, markets, hairdressers, and in private houses. ASACOs have also received training on community mobilization and marketing techniques, and some ASACOs support the social marketing agents with their sensitization activities.

Network-wide awareness raising activities have also been undertaken, such as a publicity campaigns on 22 community radio stations to increase awareness of the broad range of services provided by BlueStar franchisees.

## METHODS

We examined the performance of the MSIM BlueStar network from its inception in March 2012 until December 2015 by combining information from routine monitoring data, clinical quality audits, and client exit interviews. Routine monitoring data are collected during each visit to a social franchise and include information about the types of service and number of commodities provided. Clinical quality audits are based on observations using a standardized MSI global tool and are conducted annually; internal audits are conducted by MSIM staff on all franchisees that have trained providers in place and have been operational for at least 6 months, while external clinical audits are conducted by international MSI staff on a smaller random sample of franchises. Client exit interviews are conducted annually using an MSI global standardized interviewer-administered questionnaire to a random sample of clients following their visit; the questionnaire covers client demographics and socioeconomic status, services obtained, choice of contraceptive methods, and client experience. All data collection and analysis were conducted according to international principles of maintaining privacy and confidentiality of personal information.

In line with previous analyses of the MSI BlueStar network,^4^ results are presented under the 4 intended outputs of the MSI results framework: **access, efficiency, quality, and equity:**

- **Access:** the extent to which potential clients can reach or obtain services regardless of geographic or cultural barriers to access
- **Efficiency:** how inputs (financial, human, technical) are used to maximize output
- **Quality:** the degree to which a provider or facility meets certain objectives and perceived levels of expectations of health care delivery standards
- **Equity:** the extent to which a program ensures all clients have an equal or fair opportunity to access services

To assess **access**, consistent with previous analyses,^4^ the number of clients receiving short-acting contraceptive methods was estimated by dividing the number of commodities provided by the number of commodities needed for a full year of contraceptive protection. The estimated number of clients receiving long-acting methods (implants and IUDs) was based on the number of insertion services provided (i.e., 1 insertion was taken to be equivalent to 1 client). The number of services and commodities provided was then converted into couple-years of protection (CYPs), a standard measure of the estimated time a couple will be protected against unintended pregnancy per unit of the contraceptive method used, using standard conversion factors that account for method effectiveness and wastage.^7^ Services for condom provision and removals of implants and IUDs were excluded from estimates of client numbers and are reported separately.

**Efficiency** was measured by dividing the total number of CYPs for the MSIM BlueStar network in each year by the number of franchisees in operation at the end of each calendar year.

**Quality** was assessed using clinical audit scores and exit interview results. For clinical audit scores, the average score of audited franchisees was calculated, along with the proportion of audited franchisees scoring at or over 80%. Data on the information received by clients and client satisfaction with services received were extracted from client exit interviews; interviewed clients are asked about the information provided to them during their visit and also asked to rate their experience on a scale of 1 (very poor) to 5 (very good) on a range of questions including friendliness and respect demonstrated by providers, waiting time, and facility cleanliness.

**Equity** was assessed based on the 2015 client exit interviews, and included the proportion of clients who newly adopted a modern method of family planning (“adopters,” defined as those who had not been using any modern method of family planning during the 3 months prior to their visit), the proportion aged under 20 and 25 years of age, and the proportion living below US$1.25 and $2.50 a day as assessed via the Progress out of Poverty Index.^8^

The MSI Impact 2 model^9^ was used to estimate maternal disability-adjusted life years (DALYs) lost and the number of additional users of contraception contributed by the social franchise network from 2013 to 2015. Contraceptive services provided from 2012 to 2015 were entered into the model, along with the client profile data (proportion of clients who are adopters, continuing users of modern contraception sourced from BlueStar [“continuers”], and women who were previously served by other providers [“provider-changers”]) for 2013 to 2015. The number of additional users is the estimated number of women reached by BlueStar who contribute to growth in overall levels of contraceptive use in the country compared with 2012; the calculation accounts for some women discontinuing contraception each year and excludes the estimated number of “provider-changers” as these do not represent additional users of contraception at a national level.

Where possible, results from the MSIM BlueStar network are compared with national figures on family planning use from the 2012/13 Mali Demographic and Health Survey.^1^

## RESULTS

The MSIM BlueStar network commenced providing services in March 2012. By the end of 2015, there were 137 franchisees, up from the 70 franchisees in 2012. From March 2012 until December 2015, the MSIM BlueStar network provided 497,096 family planning and HIV-related services including 127,181 HIV counseling and 27,355 HIV testing services, along with distribution of 98,610 condoms.

The remainder of the results presented in this article focus on provision of short- and long-acting methods of contraception; condoms are excluded from measures of access as the majority of condoms provided related to HIV prevention rather than prevention of pregnancy.

### Access

#### Number of Family Planning Clients

**FIGURE 1:**
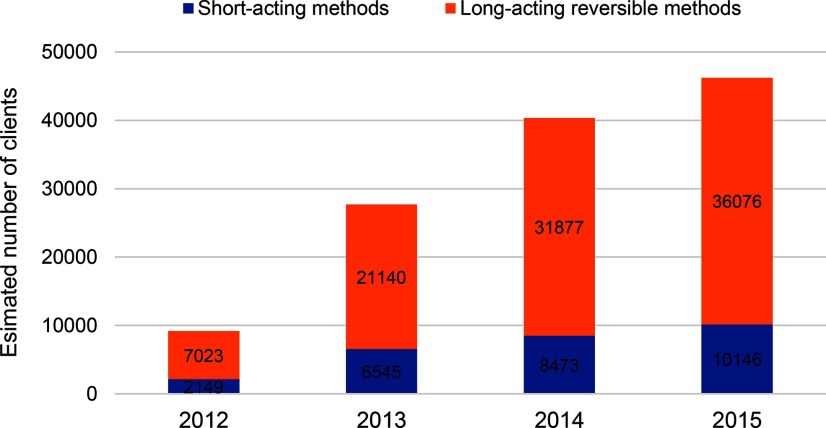
Estimated Number of Family Planning Clients Served by MSIM BlueStar Franchisees, by Year and Method Type^a^ Abbreviation: MSIM, Marie Stopes International Mali. ^a^ This figure presents estimated number of clients rather than number of services provided; if number of services were considered, services for short-acting methods would make up the majority of services provided.

The number of clients receiving voluntary family planning services at MSIM BlueStar franchisees has grown rapidly, from an estimated 9,172 clients provided with contraception in 2012 to an estimated 46,222 clients provided with contraception in 2015. Over the period March 2012 to December 2015, the MSIM BlueStar network is estimated to have provided a cumulative total of 123,429 clients with voluntary family planning services (Figure 1).

From March 2012 to December 2015, the MSIM BlueStar network provided more than 120,000 estimated clients with voluntary family planning services.

**FIGURE 2:**
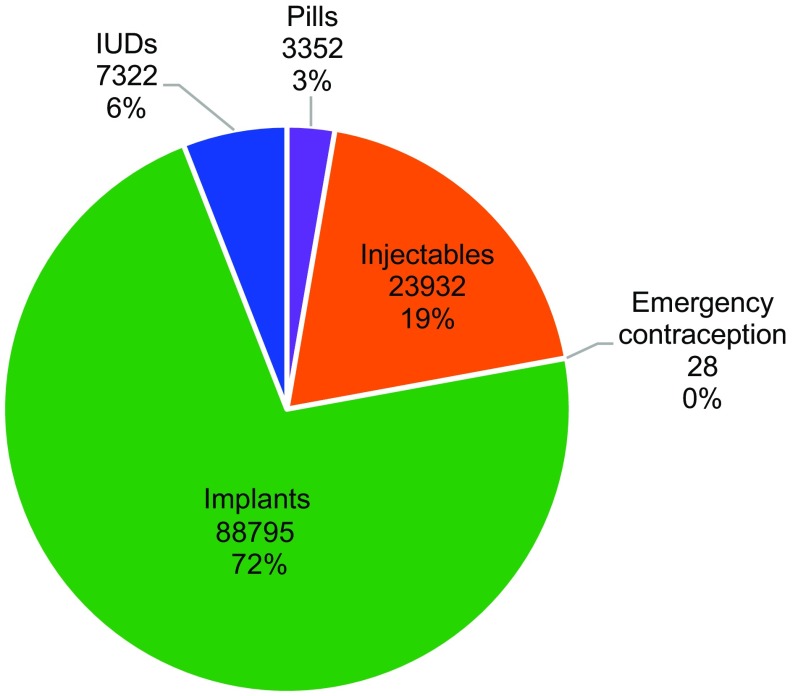
Contraceptive Method Mix^a^ Among All MSIM BlueStar Social Franchisee Family Planning Clients, 2012–2015 Abbreviations: IUDs, intrauterine devices; MSIM, Marie Stopes International Mali. ^a^ Data shown consist of number of users of each method and the percentage of method users among all family planning users.

Clients receiving long-acting reversible methods accounted for an estimated 78% of clients overall (76%–79% in each individual year; Figure 1). Implants accounted for 72% of the methods provided to clients (Figure 2) and 92% of all long-acting reversible methods provided; these proportions remained steady throughout the 4 years of franchise operation.

78% of the clients chose long-acting reversible methods.

Compared with women surveyed in the 2012/13 Mali Demographic and Health Survey who reported use of modern contraception sourced from public clinics (33%), a substantially higher proportion of clients at BlueStar franchisees were using implants (72%), and a lower proportion were using pills and injectables (Table 1). The proportion using IUDs appeared higher among clients of MSIM BlueStar franchisees, although the difference was not as substantial as for the other methods compared.

**TABLE 1.** Method Choice Among Modern Method Users, Mali DHS 2012/13 Respondents Using Modern Contraception From a Public Clinic^a^ Compared With MSIM BlueStar Franchisee Clients 2012–2015

Proportion Reporting Use of:	DHS Respondents	MSIM BlueStar Franchisee Clients				
		2012	2013	2014	2015	Overall
Pills	16.3	1.7	2.7	2.9	2.8	2.7
Injectables	44.2	21.7	20.9	18.1	19.1	19.4
Implants	33.4	72.3	70.4	72.8	72.0	71.9
IUDs	3.8	4.3	5.9	6.2	6.0	5.9

Abbreviations: DHS, Demographic and Health Survey; IUDs, intrauterine devices; MSIM, Marie Stopes International Mali.

a The users of modern contraception in the DHS are derived from the women surveyed who were married or unmarried but sexually active at the time of the survey and who reported using a modern method of contraception sourced from a public clinic. The proportions included in the table do not total 100% for these users, as the table includes only those selected methods of contraception that were available at MSIM BlueStar franchisees.

**FIGURE 3:**
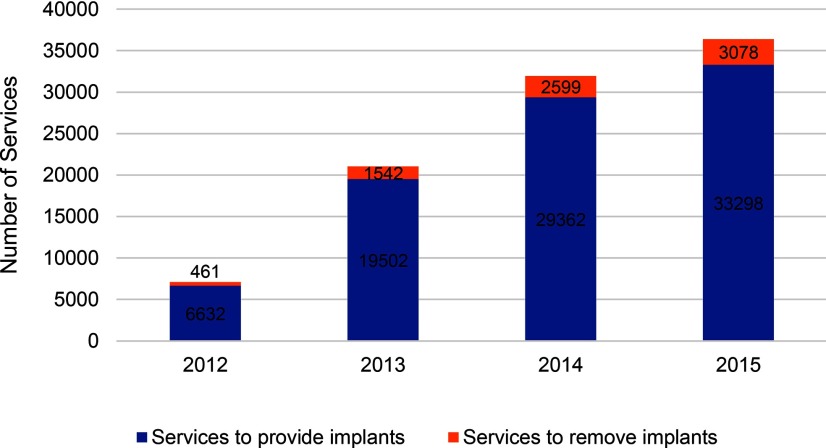
Number of Implant Provision and Removal Services at MSIM BlueStar Franchisees, by Year Abbreviation: MSIM, Marie Stopes International Mali.

**FIGURE 4:**
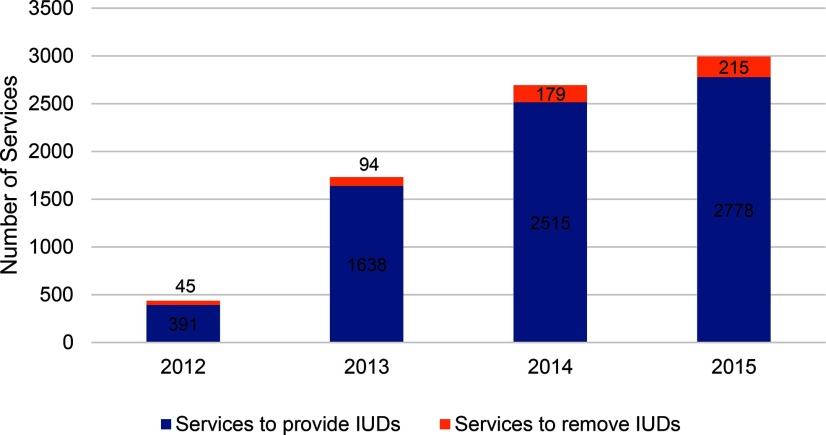
Number of IUD Provision and Removal Services at MSIM BlueStar Franchisees, by Year Abbreviations: IUD, intrauterine device; MSIM, Marie Stopes International Mali.

During the period 2012–2015, there were 8,213 services for removal of implants and IUDs at MSIM BlueStar franchisees. Removal services as a proportion of all implants and IUDs provided were 8.5% overall, increasing from 7.2% in 2012 to 9.1% in 2015 (Figure 3 and Figure 4).

#### Number of CYPs

The total number of CYPs delivered by the MSIM BlueStar network increased from 29,127 in the first 10 months of operation in 2012 to 149,282 during 2015, a more than 500% increase. Overall, the network has provided 397,952 CYPs through the end of December 2015.

### Efficiency

**FIGURE 5:**
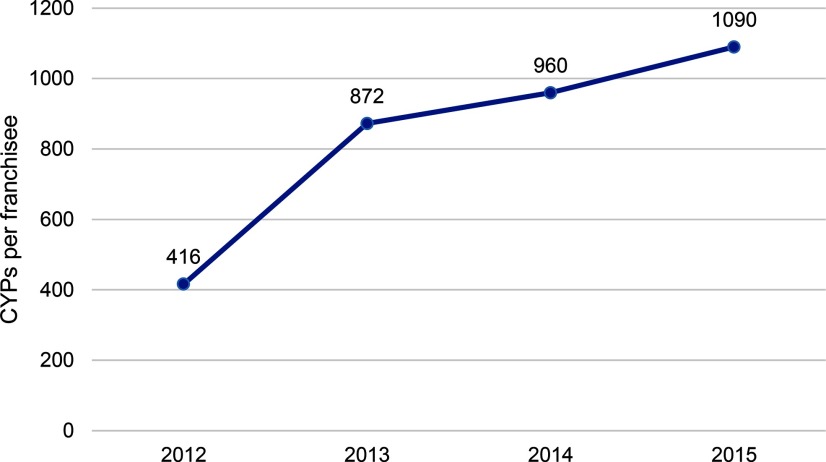
Average CYPs Provided per MSIM BlueStar Social Franchisee, per Year Abbreviations: CYPs, couple-years of protection; MSIM, Marie Stopes International Mali.

The number of MSIM BlueStar franchisees increased from 70 franchisees in 2012 to 137 franchisees in 2015. While the network almost doubled in size, the increase in franchisee outlet numbers does not account solely for the increase in estimated client numbers or CYPs. Efficiency, as measured by the number of CYPs generated by each franchisee each year, increased from an average of 416 CYPs per franchisee in 2012 to 1,090 CYPs per franchisee in 2015 (Figure 5). This compares favorably with the annual average CYPs per franchisee globally (941 in 2013)^4^ and regionally in Africa (744 in 2015; data not shown).

The average number of CYPs generated by each franchisee each year increased from 416 in 2012 to 1,090 in 2015.

### Quality

#### Clinical Quality

The quality of clinical services provided by franchisees, as measured by internal and external audits, increased over time from 2013 to 2015 (Table 2). All franchisees included in the external audits each year scored higher than the minimum standard score of 80%, and higher than the global average of 77% of MSI franchisees reaching this minimum in 2013 and 84% reaching this minimum in 2014.^4^

**TABLE 2.** MSIM BlueStar Franchisees Clinical Quality Audit Scores, 2013 to 2015

	2013	2014	2015
No. of active franchisees	101	137	137
Internal clinical audit			
No. of franchisees audited	50	93	122
Average quality score of audited franchisees	77%	80%	88%
Proportion of audited franchisees scoring 80% or higher	40%	58%	93%
External clinical audit			
No. of franchisees audited	11	10	12
Average quality score of audited franchisees	89%	90%	94%
Proportion of audited franchisees scoring 80% or higher	100%	100%	100%

Abbreviation: MSIM, Marie Stopes International Mali.

Client exit interviews confirmed these trends of high and increasing quality, with increasing proportions of surveyed BlueStar clients receiving counseling on method side effects during their visits in 2015 compared with 2013 (Table 3). The majority (80%) of clients surveyed in 2015 reported they received counseling on side effects, felt comfortable to ask questions, received about the right level of information during their visit, and learned, on average, about 4 family planning methods during their visit. Clients at social franchisees generally reported a higher level of satisfaction with clinical quality of care than clients attending other MSIM service delivery channels (data not shown).

**TABLE 3.** MSIM BlueStar Franchisee Client Exit Interview Results for Quality of Services and Client Satisfaction, 2013 to 2015

	2013 (N=220)	2014 (N=208)	2015 (N=265)
Quality-related variables			
Average number of family planning methods learned about during visit	4	3	4
Received about the right level of information during visit *(not too much or too little)*	NA	74%	82%
Were counseled on a method other than the one they received	NA	NA	92%
Received counseling on side effects	50%	86%	91%
Received instructions on what to do if had problems/side effects	93%	97%	91%
Felt comfortable to ask questions	NA	96%	80%
Satisfaction-related variables			
Satisfied or very satisfied with:			
Overall experience	100%	100%	99%
Operating hours	95%	94%	89%
Facility cleanliness	93%	98%	82%
Length of waiting time after registration	93%	96%	85%
Respectfulness of staff	98%	100%	99%
Level of privacy during time with provider	97%	99%	98%

Abbreviations: MSIM, Marie Stopes International Mali; NA, not asked.

#### Client Satisfaction

Overall clients appeared satisfied with the services received at BlueStar franchisees, with 90% of those surveyed in 2015 reporting they were likely or very likely to recommend the facility to a friend, and 97% reporting they were likely or very likely to return to the facility in the future for a service.

Looking at specific aspects of visits, generally, almost all clients were satisfied with aspects such as levels of friendliness, respect on arrival, degree of privacy, and opening hours (Table 3). Although most clients surveyed in 2015 were satisfied with facility cleanliness (82%) and length of waiting time after registration (85%), this was lower than the levels of satisfaction reported previously for these aspects (98% and 96%, respectively, in 2014), which may be due to the increased number of clients in 2015. The levels of satisfaction of franchisee clients in 2015 generally was similar to, or exceeded, the level of satisfaction of clients from the other MSIM service delivery channels (data not shown).

Regarding fees, 77% of BlueStar clients surveyed in 2015 reported paying a fee, paying, on average, 680 CFA (US$1.12). Almost all those who paid a fee in 2015 (94%) were satisfied with the fee charged, an increase from 2014 when 79% reported being satisfied with the fee charged.

77% of BlueStar clients surveyed in 2015 reported paying a fee for services and most were satisfied with the fee charged.

### Equity

Half of all BlueStar franchisee clients in 2015 were under 25 years old, including just over a quarter (26%) who were under 20 years (Table 4). Two-thirds of surveyed clients were found to be living below US$2.50 a day. Over half of clients had not completed primary education and over three-quarters of clients had 1 or more living children.

**TABLE 4.** MSIM BlueStar Franchisee Client Profile From 2015 Client Exit Interview Results (N=265)

Client Characteristic	Proportion
Age	
Under 20 years	26
Under 25 years	51
Poverty	
Living below US$1.25 a day	27
Living below US$2.50 a day	65
Living below national poverty line	36
Other demographics	
Not currently married or living with partner	23
Have not completed primary education	61
Traveled 1 hour or more to reach facility	15
Use of family planning methods	
Newly adopted a method^a^	75
Continued using a method, existing MSIM client	15
Continued using a method, previously sourced from another provider	10
Changed from a short- to long-acting method	8

Abbreviation: MSIM, Marie Stopes International Mali.

a Defined as not using a modern method in the 3 months prior to their visit.

Half of all BlueStar franchisee clients in 2015 were under 25 years old and about a quarter were under 20 years.

Compared with the 2012/13 Mali DHS, a higher proportion of BlueStar franchisee clients were aged 15–19 years (26%) compared with the proportion of modern contraceptive users in this age group in Mali as a whole (10%). Compared with the global MSI BlueStar results for 2013, a greater proportion of BlueStar clients in Mali in 2015 were aged 15–19 years (26% compared with 5% globally) and lived below US$1.25 a day (27% compared with 12% globally).

Regarding use of family planning, three-quarters of BlueStar franchisee clients in 2015 reported they had not been using a modern method in the 3 months prior to their visit—that is, they are considered adopters of family planning (Table 4). One-quarter of those surveyed were continuing users of family planning; of these, 15% were users who continued to source their method from MSI-affiliated services and 10% had previously sourced their method from another provider (Table 4). Compared with the global MSI BlueStar results for 2013, a greater proportion of BlueStar clients in Mali in 2015 were adopters of family planning (75% compared with 37% globally).

### Health Outcomes

**FIGURE 6:**
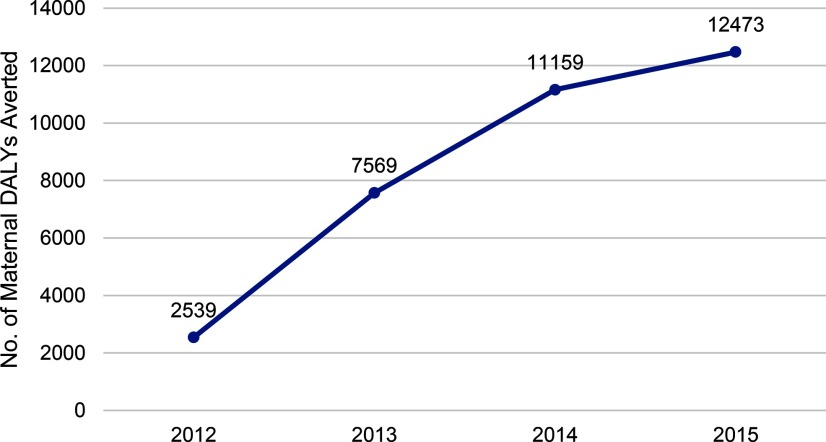
Estimated Maternal DALYs Averted Due to MSIM BlueStar Services, 2012–2015 Abbreviations: DALYs, disability-adjusted life years; MSIM, Marie Stopes International Mali.

We estimate that the services provided under BlueStar in Mali in 2015 will avert an estimated 12,473 maternal DALYs lost (Figure 6).

In terms of additionality, we estimate that BlueStar contributed 61,056 additional users of family planning in Mali over the period 2013–2015 (assuming all other providers at least maintained their baseline contributions), as well as maintaining the estimated 8,718 baseline users of modern contraception in BlueStar in 2012.

## DISCUSSION

The introduction of social franchising into the public sector in Mali has successfully increased the number of clients provided with voluntary family planning services and has reached a high proportion of young women and adopters of family planning. The intervention has proven scalable while maintaining service quality and client satisfaction. Taken together, our findings suggest that interventions that support quality supply of services while simultaneously addressing demand-side barriers, such as service pricing, can successfully create demand for a range of family planning services even in low CPR settings.

MSI's experience in Mali suggests that clinical social franchising can complement contraceptive mobile outreach service delivery models to ensure access to a broad range of voluntary family planning methods across a range of community profiles, and supports a transition from dedicated provider models to approaches more integrated with the national health system. Although not fully self-sustainable financially, we would argue that our model of training local staff and improving infrastructure and management of existing facilities is no less sustainable then many common programming approaches in the region, such as free distribution of family planning commodities. Furthermore, the fees charged to clients are retained by facilities and thus contribute to covering their ongoing operational costs.

Clinical social franchising can complement mobile outreach service delivery models to ensure access to a broad range of voluntary family planning methods.

From the Mali experience, it appears that some level of independence at subnational facilities is required to successfully franchise public-sector facilities. This includes facilities being able to self-select to join a franchise network, as well as officials who are responsible for facilities being able to enter into franchise agreements on their own, use revenue toward operating costs, and have some level of scope to reward facilities for increased quality and efficiency of services (as in Mali, where facilities retain the fees paid by clients). It is also important to ensure a functioning commodity supply chain and adequate staffing and capacity to provide a range of services.

One the challenges experienced in the implementation of the clinical social franchising model in Mali related to commodities, particularly CSCOM capacity to forecast the number of contraceptive commodities and report on stock levels. MSIM is currently in the process of building CSCOM capacity in these areas, as well as participating in processes to develop a strategic plan to improve management of reproductive health commodities nationally.

Building upon the success of the MSI social franchise model to date, Marie Stopes International Mali is currently increasing the number of franchisees in Mali and extending an adapted franchise model to rural maternity centers. The objective is to increase the range of services offered by franchisees while at the same time training traditional birth attendants (“*matrones*”) to provide implants, and to make effective referrals for contraceptive methods that they are not authorized to provide (insertion and removal of IUDs, removal of implants, provision of permanent methods). We also want to better understand the role of our pricing strategies in the success we have seen, as well as the factors underpinning our success in reaching a high proportion of young women through this service model. Not only will this learning inform future expansion of the MSI Mali BlueStar network, it will also contribute to a wider MSI strategy across all our country programs in the Sahel for social franchising, to increase access to and choice of family planning services in this high-need region.

MSIM is currently increasing the number of franchisees in Mali and extending the model to rural maternity centers.

### Limitations

Limitations of the analyses presented in this article include the estimation of client numbers from number of services and commodities provided, which may lead to over- or underestimates of true client numbers. Client numbers may also be underestimated as we did not include clients who had received family planning services such as counseling or the removal of IUDs or implants but who did not also choose to receive a modern contraceptive method at the same time. The exclusion of condom services from client and CYP estimates will have resulted in underestimates of the impact of the MSIM BlueStar franchisee network. For clinical quality audits, there were slight changes made each year to the sampling and checklists; these changes were intended to strengthen the audits based on lessons from previous years but could reduce comparability between years. Measures of client satisfaction from the exit interviews may be affected by courtesy bias. The number of additional users are a modeled estimate only and assume other providers of contraceptive services at least maintain their contribution from the baseline year of 2012. Reported results include results from the small number of privately owned practices that are part of the MSIM BlueStar franchise network (n=8 in 2015) that could not easily be excluded for analysis purposes. Finally, the model of social franchising presented in this article may not be generalizable to all public health care systems, particularly to systems where facilities are not able to generate, retain, or control their own income streams.

## CONCLUSION

Our experience in Mali suggests that our private-sector franchise model is adaptable to other types of health care facilities and can successfully increase demand for a broad range of family planning services even in low-CPR settings. Through addressing supply and demand for services, along with reducing out-of-pocket expenditure, we were able to greatly increase population access to a range of voluntary family planning services, including long-acting contraceptives. The existing network of CSCOMs provided a platform to provide high-quality family planning services at scale to rural populations, including populations most in need nof services. Similar approaches could be used in other public health systems with decentralized models, particularly where facilities have some level of independence and control over how funds are spent. MSI also operates a public franchising model in other contexts including Madagascar and Vietnam and is planning to expand the model elsewhere in the Sahel; learning regarding the relative effectiveness of our model in varied public-sector environments will inform future strengthening and scale up of the approach.

## Notes

**Peer Reviewed**
